# Characteristics of Kinematic Parameters in Decoding Intended Reaching Movements Using Electroencephalography (EEG)

**DOI:** 10.3389/fnins.2019.01148

**Published:** 2019-11-01

**Authors:** Hyeonseok Kim, Natsue Yoshimura, Yasuharu Koike

**Affiliations:** ^1^Department of Information and Communications Engineering, Tokyo Institute of Technology, Yokohama, Japan; ^2^Institute of Innovative Research, Tokyo Institute of Technology, Yokohama, Japan; ^3^Precursory Research for Embryonic Science and Technology (PRESTO), Japan Science and Technology Agency (JST), Saitama, Japan

**Keywords:** brain–machine interface (BMI), electroencephalography (EEG), classification, premovement, decoding

## Abstract

The utility of premovement electroencephalography (EEG) for decoding movement intention during a reaching task has been demonstrated. However, the kind of information the brain represents regarding the intended target during movement preparation remains unknown. In the present study, we investigated which movement parameters (i.e., direction, distance, and positions for reaching) can be decoded in premovement EEG decoding. Eight participants performed 30 types of reaching movements that consisted of 1 of 24 movement directions, 7 movement distances, 5 horizontal target positions, and 5 vertical target positions. Event-related spectral perturbations were extracted using independent components, some of which were selected via an analysis of variance for further binary classification analysis using a support vector machine. When each parameter was used for class labeling, all possible binary classifications were performed. Classification accuracies for direction and distance were significantly higher than chance level, although no significant differences were observed for position. For the classification in which each movement was considered as a different class, the parameters comprising two vectors representing each movement were analyzed. In this case, classification accuracies were high when differences in distance were high, the sum of distances was high, angular differences were large, and differences in the target positions were high. The findings further revealed that direction and distance may provide the largest contributions to movement. In addition, regardless of the parameter, useful features for classification are easily found over the parietal and occipital areas.

## Introduction

Predicting human intentions in various environments is critical in brain–machine interface research. Recently, non-invasive recordings have been widely utilized to measure brain activity due to their practicality. Various types of information have been classified, including that related to several types of movements performed during motor rehabilitation therapy (López-Larraz et al., 2014), different levels of ankle force (Jochumsen et al., 2013), standing and sitting (Bulea et al., 2014), the onset of voluntary movement (Ibáñez et al., 2014), mental arithmetic/rest (Naseer et al., 2016), finger movements (Liao et al., 2014), and braking intention (Kim et al., 2015). Of the many methods used in brain–machine interface research, electroencephalography (EEG) has been widely used because of its practical advantages. For EEG analysis, event-related potentials (ERPs), calculated by averaging brain response epochs related to events, have been used. However, the ERP does not provide all the information about an event, and the attenuated ERP amplitude makes it difficult to analyze data in a single trial (Makeig, 1993). From a frequency viewpoint, the ERP amplitude can be regarded as power in low-frequency bands. To better utilize frequency information, EEG signals have been divided based on their amplitudes in specific frequency bands, such as alpha or beta. Event-related spectral perturbations (ERSPs) (Makeig, 1993) have also been used for EEG analysis since they represent the relative frequency spectrum amplitude in the time-frequency domain.

Reaching is a fundamental and essential task in daily life. Understanding how reaching movements are represented in the brain and decoding these movements are important issues in brain–machine interface research. Several studies have attempted to decode reaching movements. For example, a study estimated the trajectory of hand movements by applying a Kalman filter to EEG data (Robinson et al., 2015), while others decoded kinematic parameters based on EEG signals during movement (Bradberry et al., 2010; Úbeda et al., 2015, 2017). Notably, the onset of a reaching movement has been detected using EEG signals obtained 1 s prior to the onset of movement (Planelles et al., 2014). EEG signals before movement onset have also been used to predict movement directions in a self-paced reaching task (Lew et al., 2014).

After a person recognizes a target, the brain may have information regarding the target that is then processed and used to develop a motor command for reaching the target. During this planning phase, proprioception is also involved in making motor commands for reaching movements (Sarlegna and Sainburg, 2009), and information about the target and arm should be integrated prior to the movement (Hoshi and Tanji, 2000). This information may not be identical to information, such as Bereitschaftspotential (Shibasaki and Hallett, 2006), observed just prior to movement execution in a self-initiated reaching task. Several previous studies have decoded brain activity just after target appearance to predict intentions regarding reaching movements. Such studies have revealed that brain signals during target recognition can be used for decoding during reaching movement planning. For classification during movement planning, EEG data are associated with higher prediction accuracy than data acquired through other modalities such as eye tracking, electrooculography, and electromyography (Novak et al., 2013). Indeed, several studies have noted that accuracies are higher than chance level in movement direction classification using EEG signals obtained at target appearance (Hammon et al., 2008; Wang and Makeig, 2009; Kim et al., 2019). For predicting peak speed and acceleration, performance is significantly better when using combined brain signals from the movement planning and execution stages than when using signals from either stage alone, suggesting that EEG signals during the planning stage can contribute to decoding (Yang et al., 2015).

These previous studies have successfully shown the utility of premovement EEG for decoding during a reaching task. However, the way the brain represents information regarding the intended target during movement preparation and what information is advantageous during decoding remain unknown. Importantly, the dorsal pathway processes visual information for a reaching task. The dorsal stream carries information from the primary visual cortex in the occipital lobe to the posterior parietal lobe (Freud et al., 2016), which has visual sensory function (Hyvärinen, 1982). The information processed in this pathway might not be identical to the parameters that researchers have classified; however, the information processed by the brain is presumably related to typical classification parameters.

Therefore, in the present study, we aimed to investigate whether parameters such as direction, distance, and positions for reaching can be decoded in premovement EEG decoding. We extracted ERSPs using EEG independent electrical sources obtained by an independent component analysis (ICA) (Makeig et al., 1996). After selecting features for classification via analysis of variance (ANOVA), we performed all possible binary classification analyses using a support vector machine with several kinds of labeling based on movements and movement parameters. In addition, we identified positions of the useful independent components (ICs) for classification; ICs refer to the electrical sources obtained by the ICA.

## Materials and Methods

### Experimental Procedure

Eight individuals (six men and two women, mean age ± standard deviation: 26.125 ± 3.27 years) participated in the experiment. All participants provided written informed consent prior to the experiment. The experimental protocol was approved by the Ethics Committees of the Tokyo Institute of Technology (ethics number: 2015062) and conducted in accordance with the ethical standards outlined in the Declaration of Helsinki.

**FIGURE 1:**
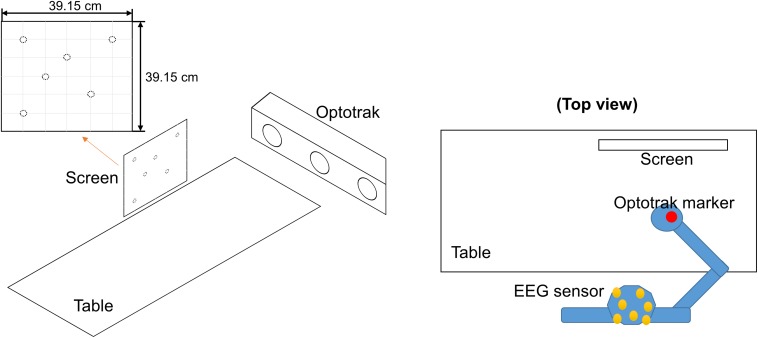
Experimental environment. The participant wore an electroencephalography (EEG) cap and sat at a desk. An Optotrak (motion sensor) marker was attached to the back of his/her right hand, and the marker position was tracked using an Optotrak device from the right side of the hand. The participant moved his/her hand on the table to move the cursor on the screen set in front of him/her. Horizontal movement on the table between right and left directions corresponded to horizontal movement on the screen, while vertical movement across the table between front and back directions corresponded to vertical movement on the screen. The initial cursor position and the target position were pseudo-randomly selected from the six positions that were decided so that all combinations from the six positions should cover all movement parameters (direction, distance, and positions for reaching) used in this research.

Figure 1 shows the experimental environment. Each participant sat in a comfortable chair adjacent to a table. The participant wore an EEG cap, and a marker for a motion sensor (Optotrak Certus, Northern Digital Inc., Waterloo, ON, Canada) was attached to the back of his/her right hand. Prior to the experiment, the participant placed his/her hand on the table to perform the required task. This position corresponded to the cursor positioned at the center of the screen. Horizontal hand movement across the table moved the cursor horizontally on the screen. However, for vertical cursor movement, the participant was required to move his/her hand vertically across the table, rather than through the air. Participants were also instructed not to touch the surface of the table during reaching movements due to the influence of friction. Thus, participants lifted their hands very slightly to perform reaching movements. The ratio between the distance of the hand and the distance of the cursor was set to 1.

**FIGURE 2:**
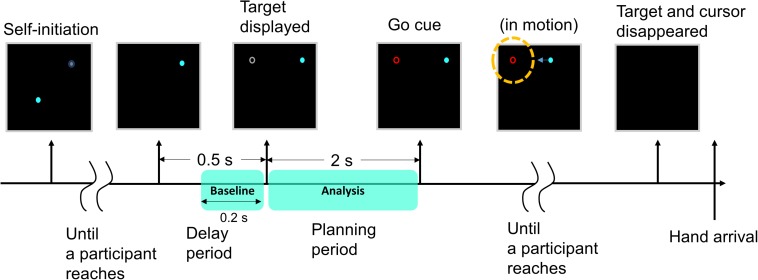
Images on the screen during a trial. At the start of the trial, an initial position indicator (a gray blurred circle) appeared at one of the six positions so that the participant moved the cursor (a blue circle) to the initial position by moving his/her hand. When the cursor reached the initial position, the indicator disappeared. After 0.5 s, a gray target appeared at one of the remaining five positions. The participant was instructed to prepare for movement execution for 2 s (planning period, premovement). When the color of the target changed to red, the participant moved his/her hand to move the cursor to the target (execution). Before the cursor completely reached the target (a yellow dotted circle in the figure), both the cursor and the target disappeared. The target and the initial cursor position were placed at one of six locations, respectively. This procedure was repeated for each trial.

Figure 2 shows the trial procedure. A target and the initial position of the cursor were placed at two of six locations, shown in Figure 1. Therefore, there are 30 different movements (6 × 5 = 30) depending on the selection of the 2 positions; the 30 movements consisted of 1 of 24 directions, 7 distances, and 5 target positions, respectively, as shown in Table 1. The participants performed the 30 different movements 10 times in one run. Then, all participants performed five runs. The trials were presented in random order, and participants were allowed to rest between runs.

**TABLE 1:** The five classes used for classification.

**Class**				
Direction	Distance	x position	y position	Each movement
(24 classes)	(7 classes)	(5 classes)	(5 classes)	
[Unit: degree]	[Unit: 1 side of the small square in Figure 1: 6.525 cm]	[Unit: 1 side of the small square in Figure 1: 6.525 cm]	[Unit: 1 side of the small square in Figure 1: 6.525 cm]	
−162, −153, −146, −135, −124, −117, −108, −90, −63,	1.414, 2.236, 3.162, 3.606, 4, 4.243, and 5.657	1, 2, 3, 4, and 5	1, 2, 3, 4, and 5	Movements 1–30
−45, −27, 0, 18, 27, 34, 45, 56, 63, 72, 90, 117, 135, 153, and 180				

At the beginning of the experiment, an initial position indicator (a gray blurred circle) appeared to set the initial position of the cursor. The participant was allowed to take a brief break and move his/her body before moving the cursor to the initial position. When the cursor (a blue circle) reached the initial position, the indicator disappeared. After 0.5 s, a gray target appeared. The participant was instructed to only look at the target and prepare for movement execution in this period and not to move any body part including the eyes (planning period). During this period, the participant planned the degree and direction of movement required to reach the target. After 2 s, the color of the target changed to red, and the participant moved his/her hand to move the cursor to the target (execution). During this period, the participant was instructed to reach the target in one attempt because feedback during movement may alter the movement trajectory (Desmurget et al., 1999). If participants were to know the final position, motor commands during the next movement may suggest an error (Tseng et al., 2007), influencing the planning phase in each trial. Therefore, before the cursor completely reached the target, both the cursor and the target disappeared. This procedure was repeated for each trial.

### Data Acquisition and Preprocessing

Hand position was measured using the motion capture system to evaluate whether the participant moved his/her hand appropriately. The Optotrak marker was attached to the back of the hand. The position data were sampled at 100 Hz. According to the international 10–20 system (Klem et al., 1999), EEG signals were measured from the following 64 electrodes using a Biosemi ActiveTwo amplifier system (Biosemi, Amsterdam, Netherlands): Fp1, Fp2, Fpz, AF3, AF4, AF7, AF8, AFz, F1, F2, F3, F4, F5, F6, F7, F8, Fz, FT7, FT8, FC1, FC2, FC3, FC4, FC5, FC6, FCz, C1, C2, C3, C4, C5, C6, Cz, T7, T8, TP7, TP8, CP1, CP2, CP3, CP4, CP5, CP6, CPz, P1, P2, P3, P4, P5, P6, P7, P8, P9, P10, Pz, PO3, PO4, PO7, PO8, POz, O1, O2, Oz, and Iz. The EEG data were sampled at 2,048 Hz.

EEGLAB (Delorme and Makeig, 2004) was used for preprocessing. The EEG signals were re-referenced to an average reference, low-pass-filtered at 1 Hz, and high-pass-filtered at 49 Hz. Due to the computational load, the data were down-sampled to 100 Hz. Epochs were extracted from the duration between the onset of the planning period and 2 s post-onset (i.e., planning period). Noisy channels, noisy trials, and trials with abnormal movement as determined via visual inspection were rejected. Then, ICA was performed using the extended Infomax algorithm in EEGLAB (Bell and Sejnowski, 1995), following which noisy ICs were rejected.

### Electroencephalogram Analysis

Using the remaining ICs, ERSPs during the planning period were calculated using EEGLAB to identify changes in the relative spectral power for each IC with respect to the baseline. The baseline interval was defined as 200 ms before the onset of premovement to the onset of premovement (when the target appeared). The ERSPs time window and the window shift sizes were 300 and 50 ms, respectively, in the planning period. The frequency range for ERSPs was 0–40 Hz, while the interval was 3.333 Hz. The planning period was 2,000 ms and the window size was 300 ms, so the period representing ERSPs values was 1,700 ms in order not to use the period beyond the planning phase. Since the window moved every 50 ms, 34 time bins were used (1,700/50 = 34).

**FIGURE 3:**
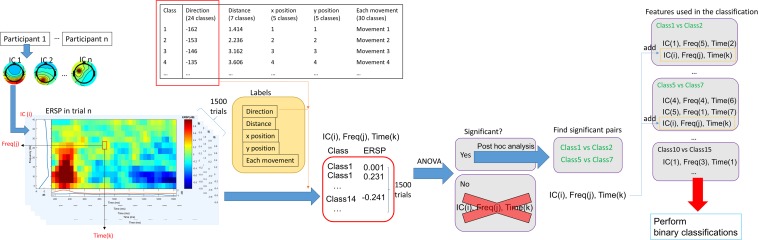
EEG signal processing. After performing an independent component analysis (ICA), ERSPs of each independent component (IC) were calculated for each trial using EEGLAB. Then, 1,500 ERSP values were obtained at each frequency at each time point in each IC (since there were 1,500 trials). We performed classification analyses using different class labeling depending on movements (i.e., 30 movements) and parameters (i.e., 24 directions, 7 distances, and 5 horizontal and vertical positions). Then, we performed an ANOVA using the 1,500 ERSP values at each frequency at each time point in each IC. If *p* > 0.05, ERSP values at that time point at that frequency in that IC were excluded from further analyses. If significant, *post hoc* analyses were performed to find significant pairs. An ERSPs at a particular time and frequency in an IC was selected as a feature for subsequent binary classifications (for significant pairs). After this procedure was completed with respect to all ICs, frequencies, and times, binary classifications using significant ERSPs were performed. ANOVA, analysis of variance; Freq, frequency; ERSP, event-related spectral perturbations.

Figure 3 shows how EEG signals were processed in this study. We obtained 1,500 ERSPs for each time point, frequency bin, and IC. Since 1 of 30 movements was performed in each trial, a trial class could be determined according to the movement. When we classified them with direction, each trial had 1 of 24 classes because the 30 movements included 24 different directions. We assigned different classes to ERSPs, in all trials, related to the parameters because each trial had 30 movements, 24 directions, 7 distances, 5 horizontal positions, and 5 vertical positions. For each parameter, a different label was assigned to a different class. MATLAB R2019a (MathWorks, Inc., Natick, MA, United States) was used to perform an ANOVA for each parameter. The different classes comprised different groups for the ANOVA. When significant differences were observed, *post hoc* analyses were performed using Tukey’s honest significant difference test to identify significantly different pairs of classes. The level of statistical significance was set to 0.05. This was conducted for all ICs, time points, and frequency bins. Thus, the analyses were designed to reveal whether the ERSPs of each IC at each time point and frequency bin was advantageous for the subsequent binary classification.

Then, all possible binary classifications were performed using all significant ERSPs as features. For example, for direction classification, the binary classifications were performed 276 times (24 choose 2) per participant when the feature for all classifications had at least 1 because there were 24 different directions. When no features were extracted from the ANOVA, classification analyses could not be performed. Therefore, we assumed that each classification had at least one feature. A support vector machine was implemented using the Statistics and Machine Learning Toolbox in MATLAB to perform binary classifications. Classification performance was assessed using five-fold cross-validation. In addition, the same classifications were performed using shuffled labels to assess whether classification performance using real labels was above the chance level. The shuffled label designation was pseudo-random and balanced.

In contrast, for movement classifications, only one vector representing the movement belonged to each class, and each classification always had two vectors to represent different kinds of movement. Thus, the parameters were investigated by comparing the two vectors. The following three values were calculated to investigate the relationship between direction and distance: angle differences, distance differences, and the sum of the distance. Since differences in the direction of the two vectors can have two values, the smaller value was selected. Regarding angle and distance differences, if accuracy for the classification where the angle difference or distance of two vectors was high, two movements could be classified by high differences in angle or distance; this suggests that the movement may be encoded in the brain by direction or distance. The sum of the distance was calculated to investigate how this relationship changes when lengths of both vectors are too short. Positions were assessed by calculating differences in target positions. If accuracy for the classification where the differences in the distance of the two targets is high, the movement may be encoded in the brain by the target position.

## Results

**FIGURE 4:**
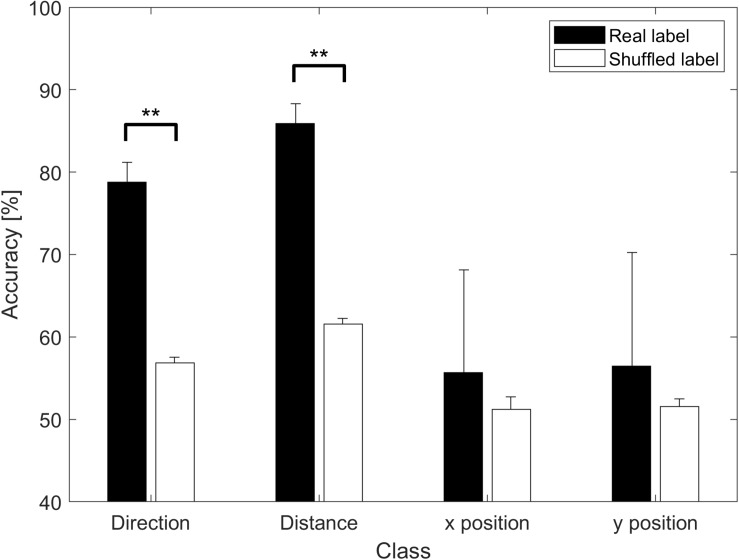
Classification accuracy based on parameters. Accuracies are represented as the means of the averaged accuracy for all possible two-class classifications across all participants. ^∗∗^*p* < 0.01. The black bar represents the result when real labels were used, while the white bar represents the result when shuffled labels were used. Significant differences were observed for direction and distance (*p* < 0.01) but not for position (*p* > 0.1).

When direction and distance were used for the class, classification accuracies significantly differed between real and shuffled labels (*p* < 0.01, paired *t*-test). However, no such differences were observed when position was used as the class (*p* > 0.1, paired *t*-test); a pseudo-random balanced shuffle was used. Figure 4 shows classification performance when different decoding parameters were used as classes. Accuracies were averaged over the classification of all possible pairs of classes, and the mean values are presented in the figure.

**FIGURE 5:**
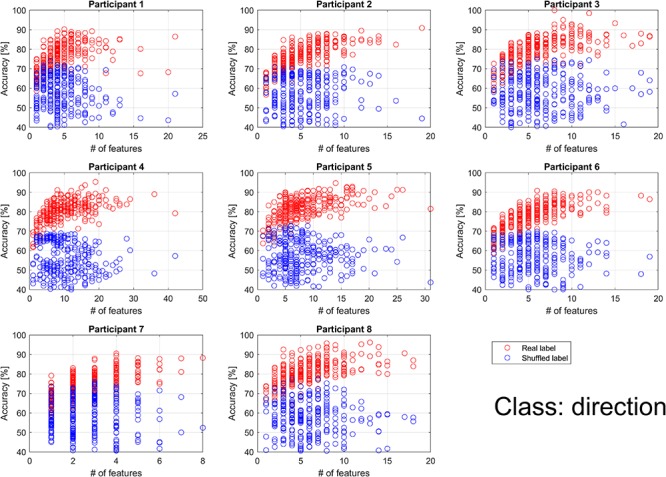
Individual classification accuracy for direction according to the number of features. Each dot represents the accuracy for each binary classification. The binary classifications were performed 276 times (24 choose 2) per participant when the feature for all classifications had at least 1. Blue circles represent the result when shuffled labels were used. Red circles represent the result when real labels were used. Performance for the real label increased in proportion to the number of features, while performance for the shuffled label did not depend on the number of features. However, performance for the real label was saturated for all participants.

For direction classification, all participants showed a higher performance than chance level. Thus, extracted features can be considered useful for the direction classification. As shown in Figure 5, performance for the real label increased in proportion to the number of features (*p* < 0.01 for all participants; *p* values were calculated for coefficients by linear regression between the number of features and the performance). For most of participants, the performance for the shuffled label did not depend on the number of features (*p* > 0.1), while data for participants 1 and 4 showed negative significant coefficients. The performance for the real label was saturated for all participants. However, saturated accuracies for all participants were similar to each other regardless of the number of features.

**FIGURE 6:**
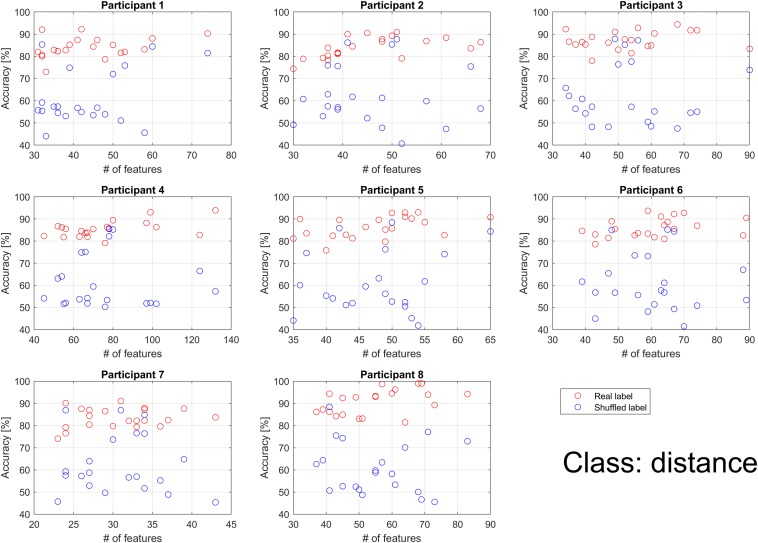
Individual classification accuracies for distance according to the number of features. Each dot represents the accuracy for each binary classification. The binary classifications were performed 21 times (7 choose 2) per participant when the feature for all classifications had at least 1. Blue circles represent the result when shuffled labels were used. Red circles represent the result when real labels were used. Performance for the real label increased in proportion to the number of features, while performance for the shuffled label did not depend on the number of features.

For distance classification, the mean accuracy was significantly higher for real labels than for shuffled labels across all participants. Thus, extracted features can be regarded as useful for the distance classification. As shown in Figure 6, some of the participants showed that performance for the real label increased in proportion to the number of features. For real labels, data for participant 2 were statistically significant (*p* < 0.01), as well as for participants 4, 5, and 8 (*p* < 0.05). Data for the other participants showed *p* > 0.1. For shuffled labels, no participants had significant coefficients (*p* > 0.1). Unlike the direction classification, accuracy did not increase exponentially, because there was no classification for which few features were utilized. In the direction classification, accuracy using shuffled labels did not exceed 80%. However, when distance was used as the class, some outliers were observed, with an accuracy of more than 80%, similar to findings observed using real labels.

**FIGURE 7:**
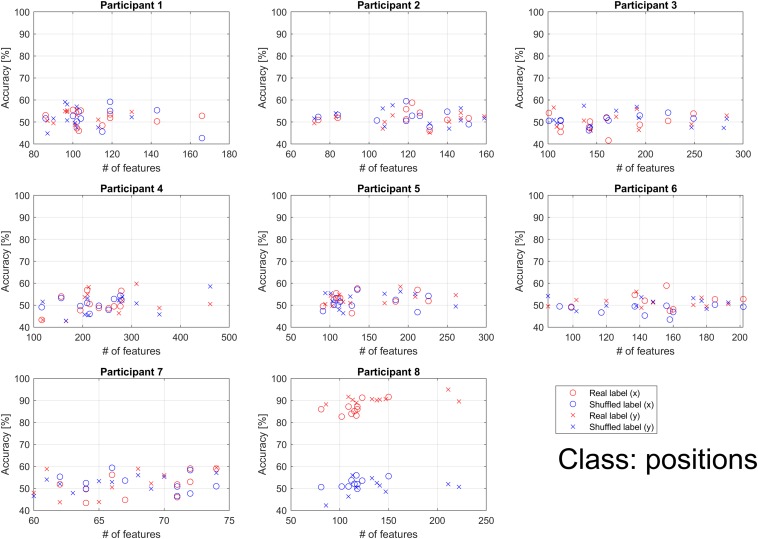
Individual classification accuracies for *x* and *y* positions according to the number of features. Each dot represents accuracy for each binary classification. The binary classifications were performed 10 times (5 choose 2) per participant when the feature for all classifications had at least 1. Blue markers represent the results when shuffled labels were used. Red markers represent the results when real labels were used. The accuracy for the horizontal (*x*) position is represented using circles, while that for the vertical (*y*) position is represented using crosses. Performance of the real and shuffled labels did not depend on the number of features. Accuracy was better than chance level in participant 8 only. For other participants, accuracy was similar to that of chance level.

For position classification, unlike the direction and distance classifications, performance for the real and shuffled labels did not depend on the number of features. As shown in Figure 7, accuracy was better than chance level in participant 8 only. Accuracy was similar to that of chance level in the other participants.

**FIGURE 8:**
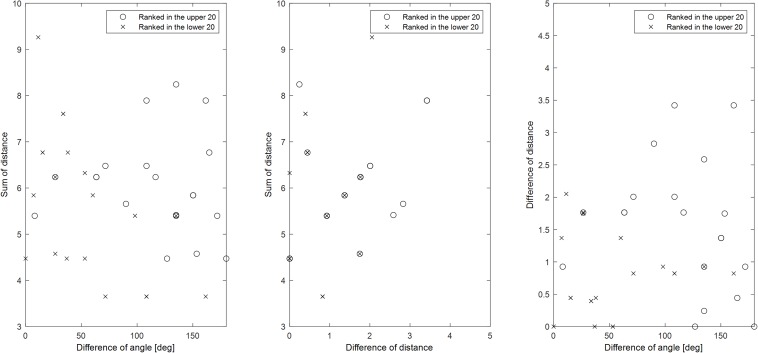
Relationships among parameters comprising two classes for classifications achieving high or low accuracy when all movements were used as different classes. Mean values across all participants were used for sorting accuracy. If the number of features for the individual classification was 0, the classification accuracy was not included in the mean accuracy calculation. When all movements were used as a different class, there were 435 classifications. Based on mean accuracy, circles represent the top 20 among these 435 classifications, while crosses represent the bottom 20. The angle difference, distance difference, and sum of distances comprising each vector representing the class were investigated. Since differences in the direction of the two vectors can have two values, the smaller value was selected (range: 0–180 degrees).

Consistent with findings observed in the direction classification, when each movement was used as a different class, performance for the real label increased in proportion to the number of features, while performance for the shuffled label did not depend on the number of features. Each classification was ranked according to the mean accuracy across all participants, following which the top and bottom 20 results were selected. If the number of features for an individual classification was 0, the classification accuracy was not included in the mean accuracy calculation. Also, the relationship between direction and distance was examined. Figure 8 shows the relationship among parameters comprising 2 classes for the top and bottom 20 classifications. Larger angle differences and sums of distance indicate greater accuracy. Larger angle differences coupled with larger distance differences are also indicative of greater accuracy. No specific relationship was observed between the distance difference and the total distance. However, when the distance difference was more than 2, high classification performance was achieved.

**FIGURE 9:**
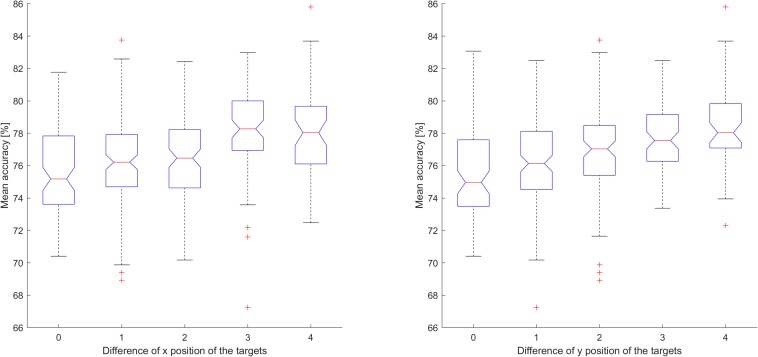
The relationship between performance and differences in the position of the target comprising two classes when each movement was used as a different class. Performance was calculated based on the mean accuracy across all participants. If the number of features for the individual classification was 0, the classification accuracy was not included in the mean accuracy calculation. In both cases, classification accuracy tended to be higher for greater differences in position (*p* < 0.01; ANOVA for both cases).

For both *x* and *y* target positions, when each movement was used as a different class, classification accuracy tended to be higher for greater differences in position (*p* < 0.01; ANOVA for both cases). Classification accuracy was lower for targets with low differences in position than for those with high differences in position. Figure 9 shows the relationship between performance and differences in target position comprising two classes when each movement was used as a different class. Accuracies for all 435 classifications were sorted according to mean accuracy.

**FIGURE 10:**
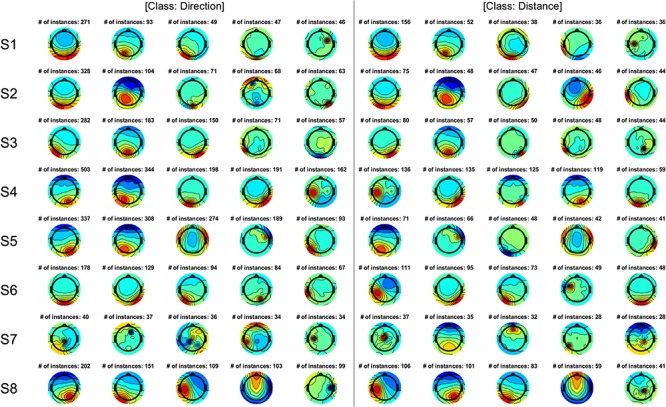
The five most frequently utilized ICs in all classifications for direction and distance. The number indicates the number of times the IC was used across all classifications. The ICs depicted were sorted according to the number of instances. IC, independent component.

**FIGURE 11:**
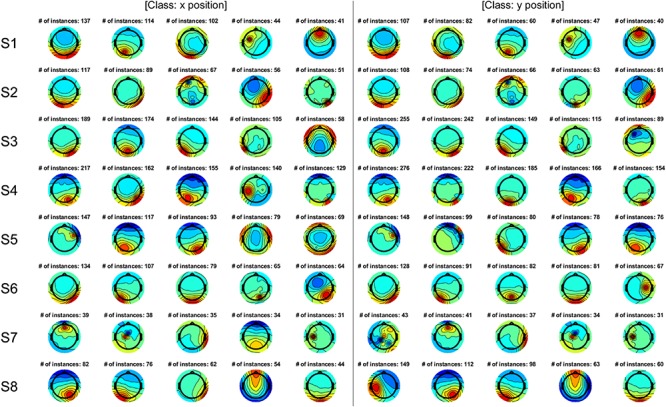
The five most frequently utilized ICs in all classifications for *x* and *y* positions. The number indicates the number of times the IC was used in all classifications. ICs were sorted according to the number of instances.

The most frequently used ICs were investigated for classification because a relationship was observed between the number of features and classification accuracy. Figure 10 shows the five most frequently utilized ICs for all classifications for direction and distance, and Figure 11 shows the five most frequently utilized ICs for classification based on position. For all parameters, ICs related to activation in the parietal and occipital areas contributed more strongly to high accuracy values than ICs related to activation in other areas and were frequently selected as significant features.

## Discussion

In the present study, we investigated the characteristics of encoded kinematic parameters of arm movement before movement execution, i.e., during movement preparation. Our analysis revealed that direction and distance classifications have some class pairs with high accuracy. Moreover, there is a relationship between these parameters (i.e., direction and distance) and the number of features extracted via the ANOVA. When each movement was used as a class, accuracy was high when the differences in the angle of the two vectors and total distance were high, the differences in the angle of the two vectors and distance were high, and the difference in the target position was high; these findings confirm that direction, distance, and position are involved in movement preparation. These observations are supported by previous findings that show the involvement of the following regions: the dorso-rostral part of Brodmann’s area 5 to combine eye position and hand position to encode the target distance (Ferraina et al., 2009); posterior parietal cortex and frontal cortical areas for sensorimotor transformation during movement planning (Andersen and Cui, 2009); and the parietal cortex, which integrates proprioceptive and visual information (Brunamonti et al., 2016).

In the classification for each kinematic parameter, accuracy was significantly higher than chance level for direction and distance, but not for position. However, this implies not that information regarding target position is useless in the prediction of the intended movement, but rather, that it is not robust, because it is easily influenced by other parameters. Previous studies have reported that movement is indeed encoded based on position (van den Dobbelsteen et al., 2001; Graziano et al., 2002; Thaler and Todd, 2009).

Our results indicate that classification accuracy for direction and distance was proportional to the number of features. However, this does not mean that the number of features directly influences classification accuracy. When position was used, classification accuracy was similar to chance level regardless of the number of features. Since we used ERSPs as a feature, a high number of features suggests that an IC showing event-related desynchronization or synchronization (ERD/ERS) in broad areas in the time-frequency domain contributes more strongly to classification accuracy than other ICs. In other words, if a feature at a specific time point or frequency bin significantly differed based on ANOVA findings but adjacent features did not, the feature may not contribute to high accuracy values. Notably, previous studies have reported ERD/ERS prior to movement execution. During the decoding of the intended movement direction, sustained ERD/ERS can be observed over the posterior parietal cortex beginning 300 ms after the directional cue (Li et al., 2012). During decoding of the intention to grasp, lift, and replace an object—which induce different kinematics—significant decreases were observed at C3 during the movement intention phase (Eilbeigi and Setarehdan, 2018). In addition, ERD has been observed at C3 prior to movement onset in the classification of different reaching movements (Shiman et al., 2017). Regions exhibiting ERD/ERS in the time-frequency domain in these previous studies exhibited values larger than each frequency bin and window shift in the present study. As these large areas were related to decoding in previous studies, the high number of features identified in our study may also be related to decoding.

For the classification of distance using shuffled labels, some classification accuracies were high, as shown in Figure 6. Upon further investigation, we observed that all outliers with an accuracy of more than 70% were related to 1 class whose distance was 2.236. Only this class had 10 different directions, while the remaining classes had 2 or 4 different directions. Therefore, despite the use of shuffled labels to avoid the influence of distance, shuffled labels were classified by direction.

As shown in Figure 10, ICs related to activation in the parietal and occipital areas contributed more strongly to high accuracy values than ICs related to activation in other areas. The posterior parietal cortex is involved in movement preparation and intention (Snyder et al., 1997; Cui and Andersen, 2007); also, this area has been used to predict intended movement direction in previous work (Wang and Makeig, 2009). In addition, motor intention increases activation in the parietal cortex (Desmurget et al., 2009). Moreover, the posterior parietal cortex plays a role in visuomotor transformation (Fogassi and Luppino, 2005). Such findings support the notion that the parietal area contributed to the high accuracy values observed in our study. Activation in the occipital area also likely contributed to high accuracy values, as the target was presented visually. Thus, information regarding the target should be treated as visual information that can then be used for motor planning (i.e., via the integration of somatosensory and visual information) (Sober and Sabes, 2005).

As shown in Figure 11, although accuracy values were similar to that of chance level, consistent with findings observed for direction and distance, ICs related to parietal and occipital activation were frequently selected as significant features. This finding suggests that position can be processed similarly to direction and distance. Furthermore, the target may be coded based on both vector and position. Previous studies have reported that movements may be coded using a combination of position and vector coding (Hudson and Landy, 2012; van der Graaff et al., 2014). In accordance with these findings, our results demonstrate that parietal and occipital activation are useful for decoding.

Even when all movements were used as different classes, direction and distance were significant factors. As shown in Figure 8, when the distance sum (or distance difference) and angle difference are high, accuracy is also high. The figure also shows that, when one of these values is small, performance can be increased by increasing one of the other values. Thus, they complement each other, suggesting that vector coding is involved in movement. Figure 9 shows that greater differences in the position of the target are associated with increases in accuracy, indicating that position is also involved in movement preparation. This seems contradictory to the results presented in Figure 4 that indicate that there was no significant difference in accuracy relative to chance level when using real data. However, the low accuracy values in Figure 4 may have been induced by various directions or distances within the class, indicating that position may not be robust for classification and that vector coding may play a more important role than position coding. In accordance with this hypothesis, previous studies have also reported that vector coding contributes more strongly to movement than position coding (van der Graaff et al., 2014).

Participant 8 exhibited significantly high accuracy even for position decoding, as shown in Figure 7. Thus, for this participant, position contributed to movement preparation in a manner similar to other parameters. However, as shown in Figure 11, most of the top ICs were related to the parietal and occipital areas, as observed in other participants, indicating that these areas may not be related to the influence of position coding on movement representation. Further studies are required to determine whether and to what extent position coding contributes to predicting intended movement. Since the frontal area is also involved in movement planning (Pobric and Hamilton, 2006; Andersen and Cui, 2009), this area may play a different role than the parietal or occipital cortices (Connolly et al., 2007), necessitating additional studies to determine how other areas are involved in classification or decoding during movement preparation. In addition to an independent area, the contribution of multiple areas, such as the network of parietal and frontal areas, may be involved in the motor control of reaching movement (Battaglia-Mayer et al., 2014; Battaglia-Mayer and Caminiti, 2019); therefore, functional connectivity of the prefrontal cortex and dorsal premotor cortex (Mattia et al., 2010) could be considered. In participant 7, the top ICs were not related to the parietal and occipital areas as in other participants (Figures 10, 11). However, the ICs involved achieved accuracy values similar to those observed in other participants, suggesting that other areas contribute to high accuracy. Furthermore, this finding suggests that information processed in the central area in participant 7 may be similar to that processed in the parietal/occipital area in other participants. Although future studies should aim to verify which type of information contributes most strongly to high classification accuracy, our findings indicate that the parietal and occipital areas play a key role and that direction and distance are advantageous for predicting intended movement.

In the current study, we calculated ERSPs of ICs at specific times and frequencies. As our approach consisted of determining significant features for each classification, this method did not reflect the fundamental differences between participant 8 and the others. Thus, an intimately linked relationship between features, including those that were not significant in this study, should be investigated. Also, connectivity between ICs should be evaluated in further studies.

## Conclusion

In the present study, we investigated the type of information the brain represents regarding the intended target during movement preparation, and what information is useful for predicting the intended movement. Our results indicated that, when each movement (i.e., pairs of the target and the initial position) was used as a labeling class, direction, distance, and position were distinguishable movement parameters for classification. However, when we classified data based on each movement parameter, only participant 8 exhibited significantly high accuracy values for the position. Thus, our findings indicate that direction and distance may contribute most strongly to the intended movement. Regardless of the parameter, our findings also demonstrate that useful features for classification are easily found over the parietal and occipital areas.

## Data Availability Statement

All datasets generated for this study are included in the article/supplementary material.

## Ethics Statement

The studies involving human participants were reviewed and approved by the Ethics Committees of the Tokyo Institute of Technology (ethics number: 2015062). The patients/participants provided their written informed consent to participate in this study.

## Author Contributions

HK designed and performed the experiment and drafted the manuscript. NY supported the experiment and analyzed the data. YK analyzed the data, contributed to the manuscript, and supervised the research.

## Conflict of Interest

The authors declare that the research was conducted in the absence of any commercial or financial relationships that could be construed as a potential conflict of interest.

## References

[B1] Andersen R. A. Cui H. (2009). Intention, action planning, and decision making in parietal-frontal circuits. *Neuron* 63 568–583. 10.1016/j.neuron.2009.08.028 19755101

[B2] Battaglia-Mayer A. Buiatti T. Caminiti R. Ferraina S. Lacquaniti F. Shallice T. (2014). Correction and suppression of reaching movements in the cerebral cortex: physiological and neuropsychological aspects. *Neurosci. Biobehav. Rev.* 42 232–251. 10.1016/j.neubiorev.2014.03.002 24631852

[B3] Battaglia-Mayer A. Caminiti R. (2019). Corticocortical systems underlying high-order motor control. *J. Neurosci.* 39 4404–4421. 10.1523/JNEUROSCI.2094-18.2019 30886016 PMC6554627

[B4] Bell A. J. Sejnowski T. J. (1995). An information-maximization approach to blind separation and blind deconvolution. *Neural Comput.* 7 1129–1159. 10.1162/neco.1995.7.6.1129 7584893

[B5] Bradberry T. J. Gentili R. J. Contreras-Vidal J. L. (2010). Reconstructing three-dimensional hand movements from noninvasive electroencephalographic signals. *J. Neurosci.* 30 3432–3437. 10.1523/JNEUROSCI.6107-09.2010 20203202 PMC6634107

[B6] Brunamonti E. Genovesio A. Pani P. Caminiti R. Ferraina S. (2016). Reaching-related Neurons in superior parietal area 5: influence of the target visibility. *J. Cogn. Neurosci.* 28 1828–1837. 10.1162/jocn_a_01004 27378332

[B7] Bulea T. C. Prasad S. Kilicarslan A. Contreras-Vidal J. L. (2014). Sitting and standing intention can be decoded from scalp EEG recorded prior to movement execution. *Front. Neurosci.* 8:376. 10.3389/fnins.2014.00376 25505377 PMC4243562

[B8] Connolly J. D. Goodale M. A. Cant J. S. Munoz D. P. (2007). Effector-specific fields for motor preparation in the human frontal cortex. *Neuroimage* 34 1209–1219. 10.1016/j.neuroimage.2006.10.001 17134914

[B9] Cui H. Andersen R. A. (2007). Posterior parietal cortex encodes autonomously selected motor plans. *Neuron* 56 552–559. 10.1016/j.neuron.2007.09.031 17988637 PMC2651089

[B10] Delorme A. Makeig S. (2004). EEGLAB: an open source toolbox for analysis of single-trial EEG dynamics including independent component analysis. *J. Neurosci. Methods* 134 9–21. 10.1016/j.jneumeth.2003.10.009 15102499

[B11] Desmurget M. Epstein C. M. Turner R. S. Prablanc C. Alexander G. E. Grafton S. T. (1999). Role of the posterior parietal cortex in updating reaching movements to a visual target. *Nat. Neurosci.* 2 563–567. 10.1038/9219 10448222

[B12] Desmurget M. Reilly K. T. Richard N. Szathmari A. Mottolese C. Sirigu A. (2009). Movement intention after parietal cortex stimulation in humans. *Science* 324 811–813. 10.1126/science.1169896 19423830

[B13] Eilbeigi E. Setarehdan S. K. (2018). Detecting intention to execute the next movement while performing current movement from EEG using global optimal constrained ICA. *Comput. Biol. Med.* 99 63–75. 10.1016/j.compbiomed.2018.05.024 29890509

[B14] Ferraina S. Brunamonti E. Giusti M. A. Costa S. Genovesio A. Caminiti R. (2009). Reaching in depth: hand position dominates over binocular eye position in the rostral superior parietal lobule. *J. Neurosci.* 29 11461–11470. 10.1523/JNEUROSCI.1305-09.2009 19759295 PMC6665750

[B15] Fogassi L. Luppino G. (2005). Motor functions of the parietal lobe. *Curr. Opin. Neurobiol.* 15 626–631. 10.1016/j.conb.2005.10.015 16271458

[B16] Freud E. Plaut D. C. Behrmann M. (2016). ‘What’ is happening in the dorsal visual pathway. *Trends Cogn. Sci.* 20 773–784. 10.1016/j.tics.2016.08.003 27615805

[B17] Graziano M. Taylor C. Moore T. (2002). Complex movements evoked by microstimulation of precentral cortex. *Neuron* 34 841–851. 10.1016/s0896-6273(02)00698-0 12062029

[B18] Hammon P. S. Makeig S. Poizner H. Todorov E. De Sa V. R. (2008). Predicting reaching targets from human EEG. *IEEE Signal. Process. Mag.* 25 69–77. 10.1109/msp.2008.4408443

[B19] Hoshi E. Tanji J. (2000). Integration of target and body-part information in the premotor cortex when planning action. *Nature* 408 466–470. 10.1038/35044075 11100727

[B20] Hudson T. E. Landy M. S. (2012). Motor learning reveals the existence of multiple codes for movement planning. *J. Neurophysiol.* 108 2708–2716. 10.1152/jn.00355.2012 22933728 PMC3545118

[B21] Hyvärinen J. (1982). Posterior parietal lobe of the primate brain. *Physiol. Rev.* 62 1060–1129. 10.1152/physrev.1982.62.3.1060 6806834

[B22] Ibáñez J. Serrano J. del Castillo M. D. Monge-Pereira E. Molina-Rueda F. Alguacil-Diego I. (2014). Detection of the onset of upper-limb movements based on the combined analysis of changes in the sensorimotor rhythms and slow cortical potentials. *J. Neural Eng.* 11:056009. 10.1088/1741-2560/11/5/056009 25082789

[B23] Jochumsen M. Niazi I. K. Mrachacz-Kersting N. Farina D. Dremstrup K. (2013). Detection and classification of movement-related cortical potentials associated with task force and speed. *J. Neural Eng.* 10:056015. 10.1088/1741-2560/10/5/056015 23986024

[B24] Kim H. Yoshimura N. Koike Y. (2019). Classification of movement intention using independent components of premovement EEG. *Front. Hum. Neurosci.* 13:63. 10.3389/fnhum.2019.00063 30853905 PMC6395380

[B25] Kim I. H. Kim J. W. Haufe S. Lee S. W. (2015). Detection of braking intention in diverse situations during simulated driving based on EEG feature combination. *J. Neural Eng.* 12:016001. 10.1088/1741-2560/12/1/016001 25426805

[B26] Klem G. H. Lüders H. O. Jasper H. Elger C. (1999). The ten-twenty electrode system of the international federation. *Electroencephalogr. Clin. Neurophysiol.* 52 3–6. 10590970

[B27] Lew E. Y. Chavarriaga R. Silvoni S. Millán Jdel R. (2014). Single trial prediction of self-paced reaching directions from EEG signals. *Front. Neurosci.* 8:222. 10.3389/fnins.2014.00222 25136290 PMC4117993

[B28] Li J. Wang Y. Zhang L. Jung T. P. (2012). Combining ERPs and EEG spectral features for decoding intended movement direction. *Conf. Proc. IEEE Eng. Med. Biol. Soc.* 2012 1769–1772. 10.1109/EMBC.2012.6346292 23366253

[B29] Liao K. Xiao R. Gonzalez J. Ding L. (2014). Decoding individual finger movements from one hand using human EEG signals. *PLoS One* 9:e85192. 10.1371/journal.pone.0085192 24416360 PMC3885680

[B30] López-Larraz E. Montesano L. Gil-Agudo Á. Minguez J. (2014). Continuous decoding of movement intention of upper limb self-initiated analytic movements from pre-movement EEG correlates. *J. Neuroeng. Rehabil.* 11:153. 10.1186/1743-0003-11-153 25398273 PMC4247645

[B31] Makeig S. (1993). Auditory event-related dynamics of the EEG spectrum and effects of exposure to tones. *Electroencephalogr. Clin. Neurophysiol.* 86 283–293. 10.1016/0013-4694(93)90110-h 7682932

[B32] Makeig S. Bell A. J. Jung T.-P. Sejnowski T. J. (1996). “Independent component analysis of electroencephalographic data,” in *Advances in Neural Information Processing Systems*, eds Touretzky D. Mozer M. Hasselmo M. (Cambridge, MA: MIT Press), 145–151.

[B33] Mattia M. Ferraina S. Del Giudice P. (2010). Dissociated multi-unit activity and local field potentials: a theory inspired analysis of a motor decision task. *Neuroimage* 52 812–823. 10.1016/j.neuroimage.2010.01.063 20100578

[B34] Naseer N. Qureshi N. K. Noori F. M. Hong K. S. (2016). Analysis of different classification techniques for two-class functional near-infrared spectroscopy-based brain-computer interface. *Comput. Intell. Neurosci.* 2016:5480760. 10.1155/2016/5480760 27725827 PMC5048089

[B35] Novak D. Omlin X. Leins-Hess R. Riener R. (2013). Predicting targets of human reaching motions using different sensing technologies. *IEEE Trans. Biomed. Eng.* 60 2645–2654. 10.1109/tbme.2013.2262455 23674417

[B36] Planelles D. Hortal E. Costa A. Ubeda A. Iáez E. Azorín J. M. (2014). Evaluating classifiers to detect arm movement intention from EEG signals. *Sensors* 14 18172–18186. 10.3390/s141018172 25268915 PMC4239925

[B37] Pobric G. Hamilton A. F. (2006). Action understanding requires the left inferior frontal cortex. *Curr. Biol.* 16 524–529. 10.1016/j.cub.2006.01.033 16527749

[B38] Robinson N. Guan C. Vinod A. P. (2015). Adaptive estimation of hand movement trajectory in an EEG based brain-computer interface system. *J. Neural Eng.* 12:066019. 10.1088/1741-2560/12/6/066019 26501230

[B39] Sarlegna F. R. Sainburg R. L. (2009). The roles of vision and proprioception in the planning of reaching movements. *Adv. Exp. Med. Biol.* 629 317–335. 10.1007/978-0-387-77064-2_16 19227507 PMC3709263

[B40] Shibasaki H. Hallett M. (2006). What is the bereitschaftspotential? *Clin. Neurophysiol.* 117 2341–2356. 10.1016/j.clinph.2006.04.025 16876476

[B41] Shiman F. López-Larraz E. Sarasola-Sanz A. Irastorza-Landa N. Spüler M. Birbaumer N. (2017). Classification of different reaching movements from the same limb using EEG. *J. Neural Eng.* 14:046018. 10.1088/1741-2552/aa70d2 28467325

[B42] Snyder L. H. Batista A. P. Andersen R. A. (1997). Coding of intention in the posterior parietal cortex. *Nature* 386 167–170. 10.1038/386167a0 9062187

[B43] Sober S. J. Sabes P. N. (2005). Flexible strategies for sensory integration during motor planning. *Nat. Neurosci.* 8 490–497. 10.1038/nn1427 15793578 PMC2538489

[B44] Thaler L. Todd J. T. (2009). The use of head/eye-centered, hand-centered and allocentric representations for visually guided hand movements and perceptual judgments. *Neuropsychologia* 47 1227–1244. 10.1016/j.neuropsychologia.2008.12.039 19428386

[B45] Tseng Y. Diedrichsen J. Krakauer J. W. Shadmehr R. Bastian A. J. (2007). Sensory prediction errors drive cerebellum-dependent adaptation of reaching. *J. Neurophysiol.* 98 54–62. 10.1152/jn.00266.2007 17507504

[B46] Úbeda A. Azorín J. M. Chavarriaga R. Millán R. J. D. (2017). Classification of upper limb center-out reaching tasks by means of EEG-based continuous decoding techniques. *J. Neuroeng. Rehabil.* 14:9. 10.1186/s12984-017-0219-0 28143603 PMC5286813

[B47] Úbeda A. Hortal E. Iáñez E. Perez-Vidal C. Azorín J. M. (2015). Assessing movement factors in upper limb kinematics decoding from EEG signals. *PLoS One* 10:e0128456. 10.1371/journal.pone.0128456 26020525 PMC4447410

[B48] van den Dobbelsteen J. J. Brenner E. Smeets J. B. (2001). Endpoints of arm movements to visual targets. *Exp. Brain Res.* 138 279–287. 10.1007/s002210100689 11460766

[B49] van der Graaff M. C. Brenner E. Smeets J. B. (2014). Misjudgment of direction contributes to curvature in movements toward haptically defined targets. *J. Exp. Psychol. Hum. Percept. Perform.* 40 802–812. 10.1037/a0034843 24364706

[B50] Wang Y. Makeig S. (2009). “Predicting intended movement direction using EEG from human posterior parietal cortex,” in *Foundations of Augmented Cognition. Neuroergonomics and Operational Neuroscience. FAC 2009. Lecture Notes in Computer Science*, eds Schmorrow D. D. Estabrooke I. V. Grootjen M. (Berlin: Springer), 437–446. 10.1007/978-3-642-02812-0_52

[B51] Yang L. Leung H. Plank M. Snider J. Poizner H. (2015). EEG activity during movement planning encodes upcoming peak speed and acceleration and improves the accuracy in predicting hand kinematics. *IEEE J. Biomed. Health Inform.* 19 22–28. 10.1109/jbhi.2014.2327635 24893371

